# Amyloid Beta Oligomers Target to Extracellular and Intracellular Neuronal Synaptic Proteins in Alzheimer's Disease

**DOI:** 10.3389/fneur.2019.01140

**Published:** 2019-11-01

**Authors:** Yu Ding, Jiahui Zhao, Xunle Zhang, Shanshan Wang, Kirsten L. Viola, Frances E. Chow, Yang Zhang, Carol Lippa, William L. Klein, Yuesong Gong

**Affiliations:** ^1^Jiangsu Key Laboratory for Functional Substance of Chinese Medicine, Department of Biopharmaceutics and Food Science, Nanjing University of Chinese Medicine, Nanjing, China; ^2^Department of Neurobiology and Neurology, Northwestern University, Evanston, IL, United States; ^3^Department of Neurology, Drexel University College of Medicine, Philadelphia, PA, United States

**Keywords:** Alzheimer's disease, synapse, postsynaptic density, soluble Aβ oligomers, α3-Na/K-ATPase, synGap, Shank3, antibody to soluble Aβ oligomers

## Abstract

**Introduction:** β-Amyloid protein (Aβ) putatively plays a seminal role in synaptic loss in Alzheimer's disease (AD). While there is no consensus regarding the synaptic-relevant species of Aβ, it is known that Aβ oligomers (AβOs) are noticeably increased in the early stages of AD, localizing at or within the synapse. In cell and animal models, AβOs have been shown to attach to synapses and instigate synapse dysfunction and deterioration. To establish the pathological mechanism of synaptic loss in AD, it will be important to identify the synaptic targets to which AβOs attach.

**Methods:** An unbiased approach using far western ligand blots has identified three synaptic proteins to which AβOs specifically attach. These proteins (p100, p140, and p260) were subsequently enriched by detergent extraction, ultracentrifugation, and CHT-HPLC column separation, and sequenced by LC-MS/MS. P100, p140, and p260 were identified. These levels of AβOs targets in human AD and aging frontal cortexes were analyzed by quantitative proteomics and western-blot. The polyclonal antibody to AβOs was developed and used to block the toxicity of AβOs. The data were analyzed with one-way analysis of variance.

**Results:** AβOs binding proteins p100, p140, and p260 were identified as Na/K-ATPase, synGap, and Shank3, respectively. α3-Na/K-ATPase, synGap, and Shank3 proteins showed loss in the postsynaptic density (PSD) of human AD frontal cortex. In short term experiments, oligomers of Aβ inhibited Na/K-ATPase at the synapse. Na/K-ATPase activity was restored by an antibody specific for soluble forms of Aβ. α3-Na/K-ATPase protein and synaptic β-amyloid peptides were pulled down from human AD synapses by co-immunoprecipitation. Results suggest synaptic dysfunction in early stages of AD may stem from inhibition of Na/K-ATPase activity by Aβ oligomers, while later stages could hypothetically result from disrupted synapse structure involving the PSD proteins synGap and Shank3.

**Conclusion:** We identified three AβO binding proteins as α3-Na/K-ATPase, synGap, and Shank3. Soluble Aβ oligomers appear capable of attacking neurons via specific extracellular as well as intracellular synaptic proteins. Impact on these proteins hypothetically could lead to synaptic dysfunction and loss, and could serve as novel therapeutic targets for AD treatment by antibodies or other agents.

## Introduction

Alzheimer's disease (AD) is a progressive neurodegenerative disorder that is the leading cause of dementia. Its pathology is characterized classically by amyloid plaques, neurofibrillary tangles, inflammation, oxidative damage, synapse loss, and selective neuron death (1–3). Extensive genetic, neuropathological, and biochemical data have established a critical pathogenic role for the amyloid β-peptide (Aβ) (2). Multiple unsuccessful clinical trials, however, have generated skepticism regarding the pathogenic role of Aβ found in amyloid plaques. As an alternative to amyloid plaques, considerable attention has become focused on soluble Aβ oligomers (AβOs), which appear to a more toxic and disease-relevant form of Aβ (4–9). AβOs, sometimes referred to as ADDLs for Amyloid-β Derived Diffusible Ligands (10), accumulate in AD-affected brain tissue and in various transgenic AD models (4, 11, 12). Experimentally, AβOs cause memory dysfunction (8, 10, 13), inhibit LTP (10, 13, 14), and prolong LTD (13). The effects of AβOs have been observed in multiple culture and animal models, including non-human primates (4, 15, 16). AβOs appear to be both necessary and sufficient for the AD phenotype. Experimental antibodies specific for AβOs rescue memory and reduce AD neuropathology (17), while AβOs and AD neuropathology sans amyloid plaques manifest in individuals with certain FAD mutations (18, 19) and transgenic mouse models (20). Evidence overall supports the opinion that the AβO hypothesis has all but supplanted the amyloid cascade (21) and the conclusion that progressive accumulation of AβOs is a central toxic event in AD (21, 22).

AβOs in brain tissue have been found in both extracellular and intracellular compartments (23–25). Although multiple AβO species have been identified in synthetic preparations and AD brain extracts from humans and transgenic models (4, 23, 26, 27), some evidence indicates two major populations, referred to as Type 1 and Type 2 (26). Type 1 oligomers are larger (~12 mers and above) and have been hypothesized to bind synapses and instigate synapse dysfunction and deterioration (7, 23, 26, 27). The increased levels of soluble AβOs in AD brain (4) show an inverse correlation with synaptic loss (28, 29), which is regarded as the best pathological correlate of dementia (1, 30). The smaller, Type 2 oligomers (mainly dimers or trimers) are prone to fibrillogenesis and may instigate neuron death (26, 27), perhaps indirectly through effects on microglia (31). Both types of AβO reportedly occur in AD brain (4, 26, 32).

The neuronal targets AβO are not yet established. It has been proposed that AβOs could insert into membrane lipids (33) but considerable evidence indicates a more selective, adventitious interaction with proteinaceous toxin receptors (4, 8, 9, 23, 34–36). An array of candidate receptors has been put forward, including interesting targets such as prion protein (37–40), PirB (41), and mGluR (34, 40, 42–44). Most recently, two independent reports have suggested that the alpha 3 form of Na/K ATPase, which is selectively expressed in neurons (45), could be an AβO toxin receptor (38, 46).

To help answer the question of which proteins could act as AβO toxin receptors, the current study takes an unbiased approach by using AβO ligand blots of human brain extracts followed by LC-MS/MS identification of bands isolated from the blotted gels. New data support our earlier observation (4) that in ligand blots, AβOs attach to three synaptic proteins of relatively high molecular weight: p100, p140, and p260. These AβO targets have been identified as α3-Na/K-ATPase, synGap, and Shank3, respectively. Results indicate that potential toxin receptors for AβOs exist both inside and outside synaptic terminals.

## Materials and Methods

### Materials

Amyloid β-protein (1–42) was obtained from American Bachem. Ham's F-12 medium phenol red-free was from Caisson Labs. Hibernate E was from Thermo Fisher Scientific. Neurobasal, horse serum, and B27 supplements were from Invitrogen. Unless otherwise indicated, chemicals and reagents were from Sigma-Aldrich. The bicinchoninic acid (BCA) protein assays, fast silver stain kit, and cell lysis buffer for Western were from Beyotime. The SDS-PAGE gels (16.5% acrylamide, Tris-Tricine) were obtained from Bio-Rad. Adenosinetriphosphatase assay kit was obtained from Nanjing Jiancheng Bioengineering Institute. 6E10 antibody was from BioLegend. Anti-α1, 2 Na/K-ATPase antibodies were from Merck Millipore, and Anti-α3 Na/K-ATPase antibody was from Santa Cruz.

### Human AD and Mice Brain Tissues

Human brain tissues were obtained at autopsy in the postmortem period (5.3 ± 0.9 h) from 5 patients diagnosed clinically and histopathologically with AD (79.7 ± 2.7 years) and from 5 age-matched controls in the postmortem period (5.0 ± 1.0 h) with no clinical or morphologic evidence of brain pathology (81.4 ± 2.0 years). The ages and PMDs of cases were not significantly different between the AD and control groups (47). All tissues were obtained from the DUCOM Memory Disorders brain bank.

3 × Tg mice brain tissues were from Dr. Robert A. Nicholes lab at Drexel University College of Medicine. APP/PS1 mice were purchased from the animal center at Nanjing University, China.

### Membrane Preparation

All manipulations of adult mice and rat tissues, including brain cortex, heart, kidney, and others, were performed at 4°C according to our previous protocol (4). All tissues were homogenized in 20 vol of buffer A (PBS, pH 7.4, 0.32 M sucrose, 50 mM Hepes, 25 mM MgCl2, 0.5 mM DTT, 200 μg/ml PMSF, 2 μg/ml pepstatin A, 4 μg/ml leupeptin, 30 μg/ml benzamidine hydrochloride), and were centrifuged at 1,000 × g for 10 min. The pellet was re-homogenized in 10 vol of buffer A and centrifuged again. The combined supernatants were centrifuged at 100,000 × g for 1 h, and the pellet was used for total membrane fraction.

### Oligomers of Aβ (AβOs) Preparation

Oligomers of Aβ1–42 peptide were prepared as previously described (10, 48, 49), Briefly, Aβ1–42 was brought to 100 μM in cold Ham's F-12 medium phenol red-free, the solution vortexed, incubated at 4–8°C for 24 h, and centrifuged (14,000 × g for 10 min), the supernatant was used as AβOs.

### Anti-soluble Oligomers of Aβ Antibody Preparation

Anti-soluble Aβ antibody was prepared as previously described (15). Briefly, a New Zealand rabbit with a weight of about 2–3 kg was selected. After 1 week of adaptive feeding, the Pre-immune serum was collected before the first injection of antigen, which was stored at −20°C for subsequent experimental control. In the first immunization, the 0.15 mg AβOs antigen and the complete Freund adjuvant (CFA) were fully emulsified by volume compared with 1:1, and 10 sites were injected immediately. After every 2 weeks, the 0.08 mg AβOs antigen and the incomplete Freund adjuvant (IFA) were emulsified and booster injected into 4 sites for 5 times in total. The final immune sera against the original antigen solution to track titer in Nitrocellulose membrane by Western Blot. The total anti-soluble Aβ IgG proteins were isolated by Protein-A Sepharose column.

### Synaptosome and PSD Preparation, and Quantitative Proteomics

Synaptosome and PSD were isolated from cortical tissues (50). Briefly, tissues were homogenized in Buffer A (0.32 M Sucrose−5 mM HEPES pH 7.4, 1 mM MgCl2, 0.5 mM CaCl2, and protease inhibitors) with a Teflon homogenizer. The homogenates were centrifuged at 1400 g × 10 min. The pellets were re-homogenized in the same Buffer A and centrifuged at 700 g × 10 min. The combined supernatants were centrifuged at 13,800 g × 10 min. The pellets were used as total membrane, after further centrifuge, synaptosomes were collected from the interface between 1 and 1.15 M sucrose for analysis and PSD preparation.

Quantitative proteomic analysis was performed with iTRAQ™ Isobaric Labeling (50). Briefly, equal amounts of sample were used for trypsin digestion and labeled with iTRAQ reagents (iTRAQ-114 and iTRAQ-115 [Control] or iTRAQ-116 and iTRAQ-117 [AD], Applied Biosystems, CA). Protein identification and quantitation were performed using a ProteinPilot 2 software (Applied Biosystems, CA) integrated with IPI-human database (version 3.24) (68,020 entries). Quantification was based upon the signature peak areas (m/z: 114, 115, 116, 117) and corrected according to the manufacturer's instructions to account for isotopic overlap. Statistically significant changes were defined by the error factor and *p*-value.

### AβOs Ligand Blot

Ligand blots were based on our published procedures (4). Membrane preparations were extracted with detergent for 15 min on ice, and the solubilized proteins were separated by SDS-PAGE for 3–4 h at 120 v and transferred to nitrocellulose membrane. Blots were incubated with Tris-buffered saline (TBS.T1) containing 5% non-fat dry milk overnight, washed three times with cold F12 medium, and incubated with 10 nM AβOs for 3 h or with 0.1 mg protein per ml extract of AD frontal cortical tissues for overnight at 4–8°C. After washing away unbound material with TBST, the bound AβOs were labeled with M71/2 (1:1,000), and were visualized with enhanced chemiluminescence (Amersham Pharmacia Bioscience).

### p100, p140, and p260 Isolation and Sequencing

#### Enrichment of AβOs Binding Proteins by Detergent Treatment and Linear Sucrose Gradient Ultracentrifuge

Detergent treatment:40 mg × 6 cortex membrane protein for adult rat cortex were dissolved in 120 ml 5 mM Tris-HCl pH 9.5 containing 0.5% sodium deoxycholate for p100 enrichment, and containing 0.5% Zwittergent for p140 and p260 enrichment for 1 h at RT. Linear sucrose gradient ultracentrifuge: 10 ml 5 mM Tris-HCl pH 7.4 containing 30–60% sucrose linear gradient was prepared and induced onto the bottom of one ultracentrifuge tube. 20 ml detergent treatment solution was applied onto the top of this sucrose linear gradient. The ultracentrifuge was run for 18 h at 100,000 × g. The fractions from the top to the bottom were collected at 1 ml per fraction for p100 enrichment, and 0.5 ml per fraction for p140 and p260 enrichment. The proteins in each fraction were used to detect p100, p140, and p260 by ligand blot. The fractions containing p100 and the pellets at the bottom were collected as coarse samples of p100 and of a mixture with p140 and p260. P100 fractions were diluted with equal volume 10 mM sodium phosphate buffer pH 7.2 containing 2% SDS at RT for 1 h, and the pellets containing p140 and p260 were dissolved in 3 ml 2% SDS, and were diluted to 1% SDS by 10 mM sodium phosphate for 1 h at RT. These solutions were centrifuged at 100,000 × g for 1 h at 21°C. The supernatants were applied onto CHT-column HPLC for enrichment of p100, p140, and p260.

#### Enrichment of AβOs Receptors by CHT-Column

The supernatant, i.e., crude extracts of AβOs binding proteins, were applied onto Econo-Pac CHT-II cartridge (Bio-Rad) equilibrated with 10 mM phosphate buffer (pH7.2), 1% SDS, and 0.5 mM DTT. After washing with the equilibration buffer, chromatography was developed with a linear gradient of potassium phosphate (from 10 to 700 mM) in the same buffer. The buffers and the column were maintained at 28°C to prevent SDS precipitation. 200 μl elution fractions were dialyzed against 1% SDS 10 mM Tris-HCl pH 7.4 overnight. These fractions were concentrated to 60 μl by ultrafiltration with a Centricon filter (Amicon, 10-kDa cut-off) and were concentrated again to 25 μl by 100% PEG.

#### Identifying AβOs Binding Proteins in Fractions From the CHT-Column

Extracts of AD cortical tissue and synthetic AβOs were used for detecting p100, and p140 and p260, respectively. 75 μg proteins of rat cortex were dissolved 30 μl Electrophoresis Sample Buffer for control. The concentrated fractions were mixed with 25 μl Electrophoresis Sample Buffer. Electrophoresis conditions were 4-20% SDS-PAGE Tris-HCl gel (Bio-Rad), 120 v, 1.5 h at RT and 2.5 h in a cold room. The transfer condition was 100 v for 1 h. The nitrocellulose membrane was blocked by 5% Milk in TBS.T1 overnight, and was washed by TBS.T1 3 × 15 min at room temperature. Proteins on nitrocellulose membrane were incubated with 0.1 mg proteins per ml AD extract overnight or 10 nM sAβOs in 10 ml F12 Media for 3 h in a cold room. The nitrocellulose membranes were washed by TBS.T1 3 × 15 min at room temperature and incubated with primary antibody M71/2 1:4,000 in TBS.T1 with 5% milk for 1 h at RT. The membrane was washed by TBS.T1 3 × 15 min at RT and incubated with second antibody to rabbit Ig M71/2 1: 16,000 with 5% milk for 1 h at RT, then washed by TBS.T1 3 × 15 min at RT. The image was developed by ECL, SuperSignal West Femto Kit (Pierce 0.5 ml each reagent and 1.0 ml water).

#### LC-MS/MS

The fractions containing p100, and containing p140 and p260 were collected, and were dialyzed against 1% SDS 10 mM Tris-HCl pH 7.4 overnight. Fractions containing p100, concentrated by ultrafiltration with a Centricon filter (Amicon, 50-kDa cut-off), and fractions containing p140 and p260, by ultrafiltration with a Centricon filter (Amicon, 100-kDa cut-off), were concentrated again to 25 μl by 100% PEG. The gel containing p100, p140, and p260 proteins, compared with the bands in AβOs ligand blot, were cut out for sequencing. Proteins were digested with trypsin, peptides were analyzed by LC-MS/MS. Peptide sequences were searched by Mascot in East Lansing.

### Immunoprecipitation

Synaptosomes of AD and control frontal cortex were resuspended in RIPA buffer, the supernatants at 100,000 g for 1 h were mixed with magnetic beads which were crossed with antibody to oligomers of Aβ or to NKAα3. After that, non-specific binding proteins were washed by RIPA buffer. The proteins on the beads were released at low pH and analyzed by dot blot by 6E10 for β-amyloid peptides, and by western blot for NKA α1, α2, α3 isoforms (51).

### Na/K-ATPase Activity Assay

The neuronal membrane and synaptosomes were prepared from mice cortical tissues (4). The collected membrane fractions and synaptosomes were resuspended in buffer with 10 mM Tris-HCl, 150 mM NaCl, pH7.4 for Na/K-ATPase activity assay kit (Nanjing Jiancheng, China). The kit was used to measure inorganic phosphate released from ATP hydrolysis in the medium: 130 mM NaCl, 20 mM KCl, 3 mM MgCl2, 3 mM ATP, and 30 mM HEPES (pH 7.4 at 37°C for 10 min) by calculating Na/K-ATPase activity as the difference between total and ouabain independent ATPase. Ouabain-sensitive Na/K-ATPase activity was calculated as the difference between ATPase activity in the presence and in the absence of 1 mM ouabain (52).

### Data Analysis

For each experiment, two or three independent replicated experiments were performed. The densities of immunoblot were acquired with densitometric scan and quantified with Image J. Results were expressed as means ± SEM. The data were analyzed with one-way analysis of variance. Statistical significance was determined at *p* < 0.05.

## Results

### Binding Proteins for Oligomers of Aβ (AβOs) Were Enriched by Detergent Extraction, Ultracentrifugation, and CHT-Column HPLC Separation

Rat cortical synaptosomes were previously reported to contain three proteins that bind AβOs in far Western ligand blots, referred to as p100, p140, and p260 according to their molecular weights (4). These proteins were found in detergent-resistant membrane fractions presumably associated with rafts and post-synaptic densities (4). As a first step toward enriching p100, p140, and p260, we sought to selectively remove proteins that did not bind AβOs from the synaptosomes using various detergents. No selectivity was found for 0.1% SDS, but milder detergents (TritonX-100, octyl-glucoside, CHAPS, Zwittergent, sodium deoxycholate) released <50% of p100 and <5% of p140 and p260 (data not shown).

To enrich p100 sufficiently for LC-MS/MS analysis, we used sodium deoxycholate to first remove proteins that did not bind AβOs and then we fractionated the remaining detergent-resistant particles (which were enriched in p100) by 30–60% linear gradient sucrose ultracentrifuge.

**Figure 1:**
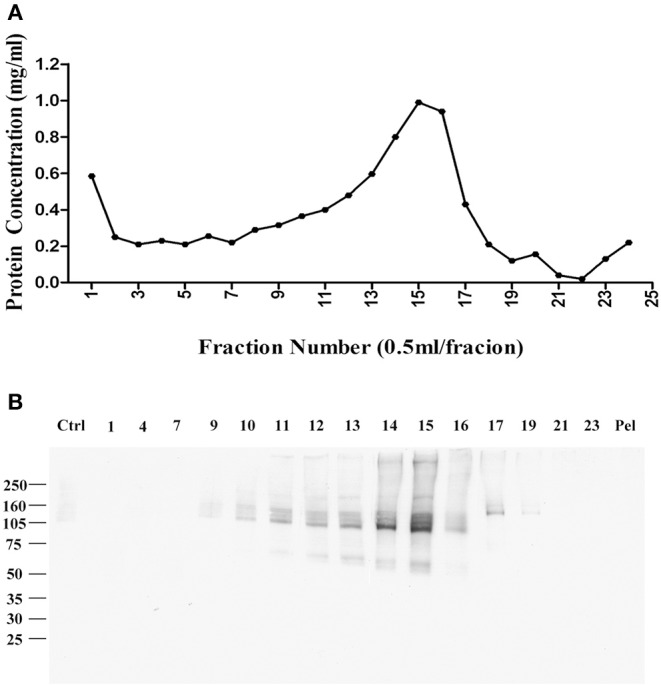
Enrichment of AβO binding protein p100 by linear sucrose gradient ultracentrifuge. Cortical membrane was resuspended in Tris-HCl buffer with cholate, which was separated by linear sucrose gradient ultracentrifugation. The fractions from the top to the bottom were collected at 1 ml per fraction, and the pellets were solubilized in 1 ml 1% SDS. **(A)** The protein concentration in each fraction. **(B)** AβO binding proteins in each fraction identified by ligand blotting. Endogenous AβO binding p100 as found primarily in fractions 14 and 15 Pel: pellets.

Fractions were collected from top to bottom, with the total protein in each fraction shown in Figure 1A. Virtually no protein was found in a pellet, indicating the detergent-resistant particles were quite small. To identify which fractions contained p100, we carried out a ligand blot using brain-derived AβOs. Binding of AβOs was identified using the AβO specific antibody M71/2 (4). Proteins in equal volumes of these fractions were separated on SDS-PAGE and transferred to nitrocellulose membrane for detection of AβO binding proteins. Figure 1B shows distribution of p100 in the separated deoxycholate-resistant fractions.

**Figure 2:**
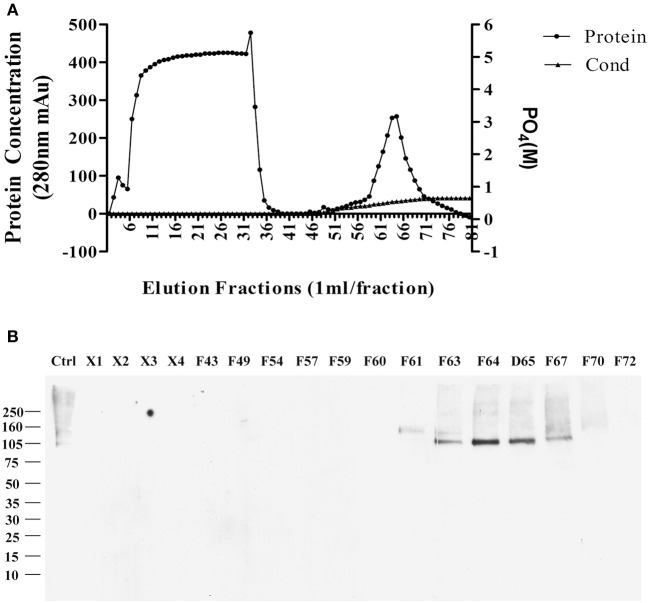
Further enrichment of AβO binding protein p100 by CHT Cartridge HPLC. Fractions from the sucrose gradient containing the highest amount of p100 (fractions 14 and 15) were treated with SDS and 1 h at 100,000 g. Filtered supernatant were applied to CHT-column and fractions washed from the column by linear gradient sodium phosphate (10–700 mM). **(A)** Relative protein abundance in fractions determined by absorbance and by conductance. **(B)** Selected fractions assayed for presence of endogenous AβO binding protein p100 by ligand blotting. Proteins in fractions X1–X4 did not bind to column and showed no AβO binding proteins. Fractions containing p100 (F63–65) were collected and dialyzed against 10 mM Trit-HCl pH 7.4, and concentrated by ultrafiltration for LC-MS/MS.

The sodium deoxycholate-resistant particles in fractions that were enriched in p100 were then fully solubilized by diluting with equal volume of 2% SDS in 10 mM phosphate buffer. The solubilized proteins were applied to a CHT-column for HPLC and eluted with sodium phosphate buffer with potassium phosphate gradient. Figure 2A shows the protein profile from this gradient. Equal volumes of the fractions were used to determine which fractions contained p100, assayed by ligand blot using AD brain-derived AβOs (Figure 2B).

Fractions containing p100 were used for LC-MS/MS analysis, with results described in the next section.

**Figure 3:**
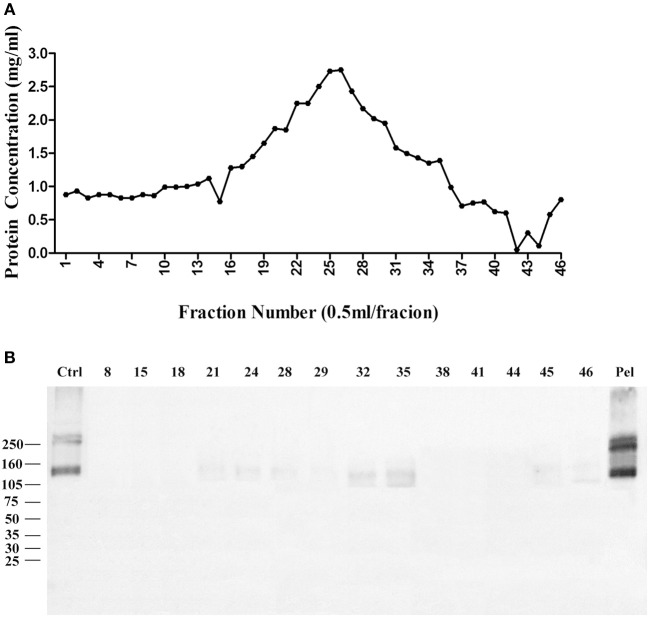
AβO binding protein p140 and p260 are not extracted from cortical membranes by Zwittergent. Cortical membranes were extracted by Zwittergent, proteins separated by ultracentrifugation, and fraction assayed by ligand blots to detect AβO binding proteins. **(A)** The protein concentration in each fraction. **(B)** AβO ligand blots for selected fractions and pellet. Results show p140 and p260 remain with the pellet of Zwittergent-extracted membranes.

We then used Zwittergent 3-12 to treat synaptosomes to remove non-specific proteins to resuspend detergent-resistant particles enriched in p140 and p260. The detergent-resistant particles containing AβO-targeted proteins were next fractioned by 30–60% linear gradient sucrose ultracentrifuge. As above, fractions were collected from top to bottom, assayed for protein, and transferred to nitrocellulose membrane for detection of AβO binding proteins by ligand blots. Protein in each fraction is shown in Figure 3A, and the ligand blots for each fraction are shown in Figure 3B. Whereas, p100 was found to be released into smaller fractions by deoxycholate (Figure 1B), the p140 and p260 binding proteins largely remained in the high-speed pellet after extraction of membranes with Zwittergent 3-12.

**Figure 4:**
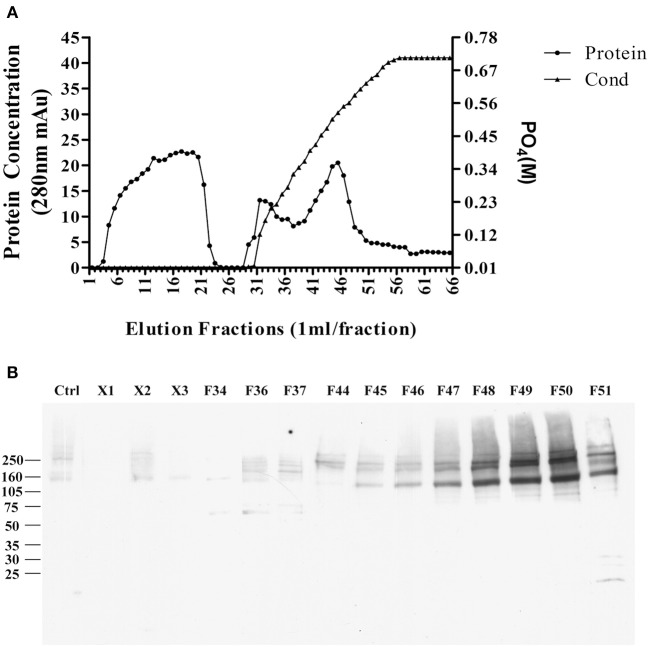
Enrichment of AβO binding proteins p140 and p260 by SDS extraction and CHT Cartridge HPLC. Zwittergent-extracted membrane pellet containing p140 and p260 (see Figure 3) was dissolved in SDS and centrifuged at 100,000 g × 1 h. The supernatant was filterd and applied to a CHT-column. Proteins were eluted by a linear gradient sodium phosphate (10–700 mM). **(A)** Protein concentration profile from column. X1, X2, and X3 were fractions that did not bind to the column. **(B)** AβO ligand blots of eluted fractions. Fractions F49 and F50 were found to contain p140 and p260 proteins. These fractions were dialyzed, and concentrated by for LC-MS/MS.

The pellet was then, solubilized in 10 mM phosphate buffer containing 2% SDS, and diluted to 1% SDS, the supernatant was applied to a CHT-column column for HPLC, and protein was eluted with sodium phosphate buffer with gradient potassium phosphate (Figure 4A). Binding proteins p140 and p260 were detected by ligand blots using synthetic AβOs and the M71/2 AβO-specific antibody, shown in Figure 4B.

As with the enriched p100 fractions, the fractions from the CHT column containing p140 and p260 were analyzed by LC-MS/MS (next section).

### Na/K-ATPase, synGap, and Shank3 Were Identified as AβOs Binding Target Proteins by LC-MS/MS

Proteins from the three AβO binding protein bands (p100, p140, and p260) were analyzed by LC-MS/MS and the outcomes listed in Table 1.

**Table 1:** Proteins were identified for p260, p140, and p100 from LC-MS/MS data.

**Protein fractions**		**Protein**	**Gi**	**Mass**	**Total score**	**Peptide matched**
p100		Na/K-ATPase α1	69,78,543	1,12,982	2,013	45
		Na/K-ATPase α3	69,78,547	1,11,664	1,688	33
		Na/K-ATPase α2	69,78,545	1,12,145	1,444	31
		ATP synthase α subunit	1,14,523	58,790	482	10
p140 p260	p140	KIAA1938 protein (Homo sapiens)	27,529,961	1,51,481	2,032	55
		Ras GTPase-activating protein, synaptic-rat	76,14,063	1,43,024	2,014	55
		syn-Gap a	1,01,22,138	1,41,981	2,014	55
	p260	Proline rich synapse associated protein 2	52,62,748	1,92,269	1,633	45

Molecular analysis of the p100 protein fraction from synaptosomes indicated the possible presence of α1, α2, and/or α3 Na/K-ATPase. Of the candidates inferred from the peptides identified by LC-MS/MS, the molecular sizes of these ATPase proteins (110 kDa) were closest to that of p100. We note that ATP synthase also is listed in Table 1 because the molecular size of its dimer is close to 100KD, and it has been reported to be a binding protein for oligomeric synuclein (53). To help resolve which of the ATPases constitute the p100 binding protein, we carried out AβO ligand blots using membranes from brain and heart (Figure 7) and blood (not shown). Heart and blood cell membranes are known to contain α1 and α2 but not α3 Na/K-ATPase (54, 55). In contrast to the results with brain samples, ligand blots of heart and blood cell membranes presented no evidence of the p100 AβO binding protein. We infer therefore that the p100 AβO binding protein of synaptosomes comprises the neuronal α3-Na/K-ATPase (56). Evidence supporting this inference is presented below.

With respect to the band containing the p140 AβO binding protein, four proteins with molecular sizes over 100 kDa were identified (Table 1). Three of the proteins were matched to synGap based on molecular size and fragment sequences (Table 1). P140 thus appears to be synGap, which is found at excitatory synapses and is associated with the NMDA receptor network in post-synaptic densities (57).

For the p260 AβO binding protein, only one protein was identified whose molecular size was close to p260 (Table 1). A sequence of 45 peptides from p260 matches with Shank3, also is a key protein involved in organizing the structure of the post-synaptic density (PSD).

### Altered Levels of α3-Na/K-ATPase, synGap, and Shank3 in PSD of Human Alzheimer's Brains

Synapses are lost in AD brains (1, 44), likely associated with the impact of AβOs, which experimentally bind synapses and cause disruption of their function and instigate their deterioration (7). Proteins identified by the LC-MS/MS results thus are relevant to AβO effects, as Na/K-ATPase is a neuronal membrane protein (56), while synGap and Shank3 proteins are in PSDs of excitatory synapse (58, 59).

**Figure 5:**
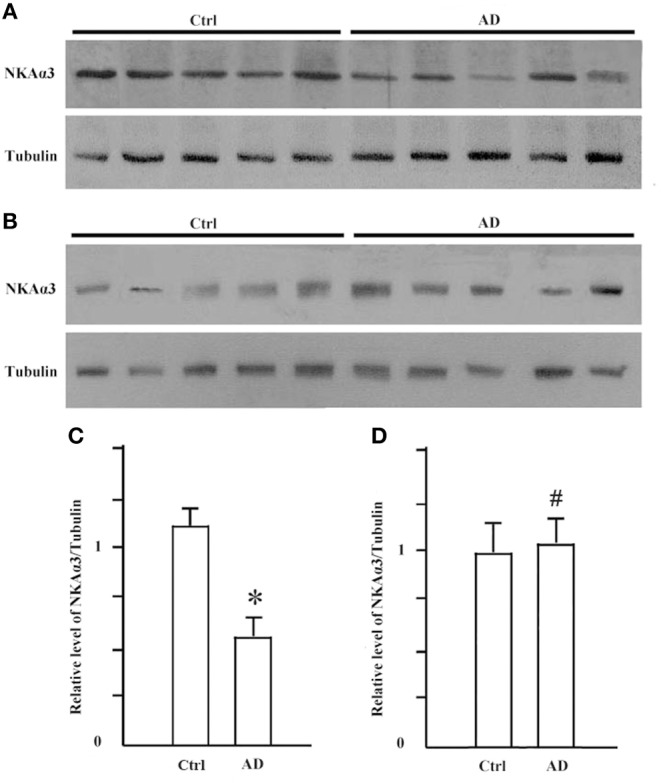
The levels of NKA α3 protein were decreased in PSD fractions of human AD cortex. Synaptosome and PSD fractions were prepared from 5 case human AD and 5 case control frontal cortical tissues. 20 μg proteins of each fraction were used for western blot. **(A)** NKA α3 proteins in PSD fractions were detected by western blot. **(B)** NKA α3 proteins in synaptosome fractions were detected by western blot. **(C)** Relative amount of NKA α3 proteins in control PSD at 1.10 ± 0.10 show decrease to 0.58 ± 0.09 in AD. Each value is expressed as mean ± SEM. *n* = 5, *p < 0.05. **(D)** Relative amount of NKA α3 proteins in synaptosome fractions show no significant difference. Each value is expressed as mean ± SEM. *n* = 5, ^#^*p* > 0.05.

It is known that synGap and Shank3 proteins are lost in human AD cortical tissues (50). Here, we analyzed the quantitative proteomic data from synaptosome and PSD fractions of human AD cortical tissues to investigate possible changes in ATPase isoforms. All three α1, 2, 3-Na/K-ATPase isoforms were in the synaptosome fraction, but only α3-Na/K-ATPase was identified in the PSD fraction. The data indicated decreased levels of α2 and α3-Na/K-ATPase proteins. Because α3-Na/K-ATPase appeared to be isoform that acted as an AβO binding protein, we further investigated the possible α3-Na/K-ATPase protein loss in PSDs of human AD brains. Synaptosome and PSD fractions were obtained from 5 human AD cortical tissues and 5 human control tissues, and α3-Na/K-ATPase protein levels in these fractions were examined by Western blots. Total α3-Na/K-ATPase protein levels did not change in the synaptosome fractions; however, in PSDs, the α3-Na/K-ATPase protein levels by 50% (Figure 5). This result confirmed the loss of α3-Na/K-ATPase protein in PSD fractions of human AD cortical brains that were analyzed by quantitative proteomics (Table 2).

**Table 2:** Na/K-ATPase in synaptosome and PSD were quantified by iTRAQ analysis.

**Fraction**	**Gene name**	***N***	**Accession IPI**	**AD116:Ctrl114**			**AD117:Ctrl 114**		
				**Ratio**	***p***	**EF**	**Ratio**	***p***	**EF**
Syn	ATP1A3	3	00788782.1	0.96	>0.05	1.20	1.08	>0.05	1.12
	ATP1A2	30	00640401.1	0.71	<0.01	1.10	0.73	<0.01	1.10
	ATP1A1	71	00006482.1	0.94	>0.05	1.17	0.99	>0.05	1.44
PSD	ATP1A3	220	00788782.1	0.57	<0.01	1.28	0.59	<0.05	1.37

*Syn, synaptosome; PSD, postsynaptic density; ATP1A3, NKAα1; ATP1A2, NKAα2; ATP1A1, NKAα1; N, the rank in the data of iTRAQ; Ctrl, Control; Ratio, protein level between AD and Control, p, p-value indicating protein significant expression in samples for identification; EF, error factor*.

### Aβ Targeted α3-Na/K-ATPase at Synapse in Human AD Brain

**Figure 6:**
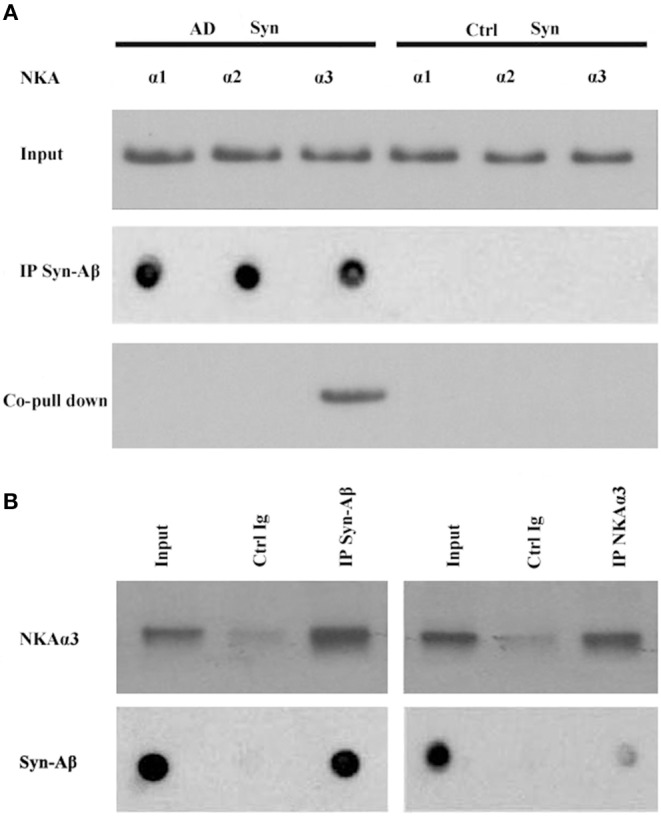
Co-IP of synaptic β-amyloid peptides and NKA α3 by AβO-antibody or NKA α3-antibody. RIPA buffer extracts of synaptosomes from human AD and control frontal cortex were incubated with magnetic beads cosslinked to antibody for AβOs or for NKAα3. Proteins released from beads were analyzed for NKA α1, α2, α3 isoforms by Western blot or for β-amyloid peptides by dot blot with 6E10. **(A)** AβO-antibody pulled down Aβ (dot blot) and NKA α3 (Western blot) from AD but not control synaptosome extracts. **(B)** Left panel: AβO antibody pulled down NKA α3 (Western blot) and Aβ peptides (dot blot) from human AD synaptosome. Negative control was carried out with magnetic beads coupled to non-specific IgG. Right panel: NAK α3 antibody pulled down NKA α3 (Western blot) and Aβ peptides (dot blot) from human AD synaptosome. Negative control carried out with non-specific IgG. Results show that NKA α3 complexes containing AβOs were present in AD but not control brain synaptosome and could be pulled down by co-IP.

Synthetic AβOs target α3-Na/K-ATPase in cultured neurons (38, 46), with AβOs causing ATPase redistribution and removal from the surface (Klein, unpublished). From human AD cortical brain tissue, soluble assemblies of Aβ preferentially bind to p100 (4). As soluble assemblies of Aβ have multiple species, we investigated further which human brain-derived species would bind synaptic proteins. We hypothesized that synaptotoxic-relevant assemblies of Aβ would bind to synaptic proteins in human AD brain. Therefore, we developed a polyclonal antibody D70 to soluble assemblies of Aβ in order to pull down synaptic Aβ assemblies from human AD cortical tissues. Results showed α3-Na/K-ATPase, but not α1, 2-Na/K-ATPase, could be co-precipitated by the D70 antibody (Figure 6). SynGap and Shank3 proteins did not show in fractions co-precipitated by the Aβ antibody (data not shown). Next, human AD synaptosomes were solubilized in RIPA buffer, the supernatants tested for the capacity of anti-α3-Na/K-ATPase to co-precipitate β-amyloid peptides. Assessment of Aβ pull down was determined by dot immunoblots using the 6E10 antibody. Results show that α3-Na/K-ATPase and synaptic Aβ were co-immunoprecipitated (Figure 6).

**Figure 7:**
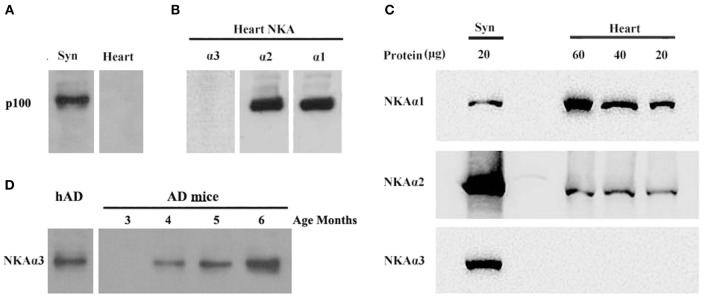
AβOs selectively bind NKA α3 in brains not in hearts of mice and at early stage of 3 × Tg AD mice. **(A)** Mouse brain synaptosome membranes and mouse heart membranes were assayed for AβO binding proteins by ligand blots. Oligomers of Aβ selectively bind p100 in brain not in heart cell membranes. **(B)** Western blots of heart cell membranes show minimal NKA α3 related to α1 and α2. **(C)** Western blots showing relative abundance of NKA α1 and α2, not α3 proteins in synaptosome compared to heart cell membranes (assayed at 20, 40, 60 μg protein). **(D)** AβO ligand blot showing the soluble AβOs, extracted from 3 × Tg mice, binding to NKA α3 (assayed 3, 4, 5, 6 months old). Overall, results show p100 in mouse also is NKA α3, which is much more abundant in brain than heart, AβOs could bind to NKA α3 in early stage of AD.

To further substantiate binding of oligomers of Aβ to α3-Na/K-ATPase, we compared α1, 2, 3-Na/K-ATPase distribution in heart cell membrane and cortical synaptosomes obtained from mouse tissue (Figure 7). In mouse, cortical synaptosomes were unique in containing all three α1, 2, 3-Na/K-ATPase proteins. Heart cells membranes showed only α1- and 2-Na/K-ATPase in Western blots. Consistent with previous results, soluble Aβ in a ligand overlay assay only bound to the cortical synapse membranes, which contained α3-Na/K-ATPase; no binding was evident when the heart cell membrane was probed (Figure 7). These results again indicated that oligomers of Aβ bind to α3 subtype of the Na/K-ATPase.

To confirm oligomers of Aβ binding to α3-Na/K-ATPase in early stage of AD development, we extracted the soluble oligomers of Aβ from the cortical tissues of 3 × Tg mice from 3 to 6 months old for AβOs ligand blot. The AβOs in extracts of cortical tissues of 3 × Tg mice obviously bound to α3-Na/K-ATPase as early as 4 months old (Figure 7D), this result suggested AβOs could alter Na/K-ATPase activity in early stage of AD.

### Anti-β-Amyloid Antibody Restores Na/K-ATPase Activity Inhibited by AβOs

**Figure 8:**
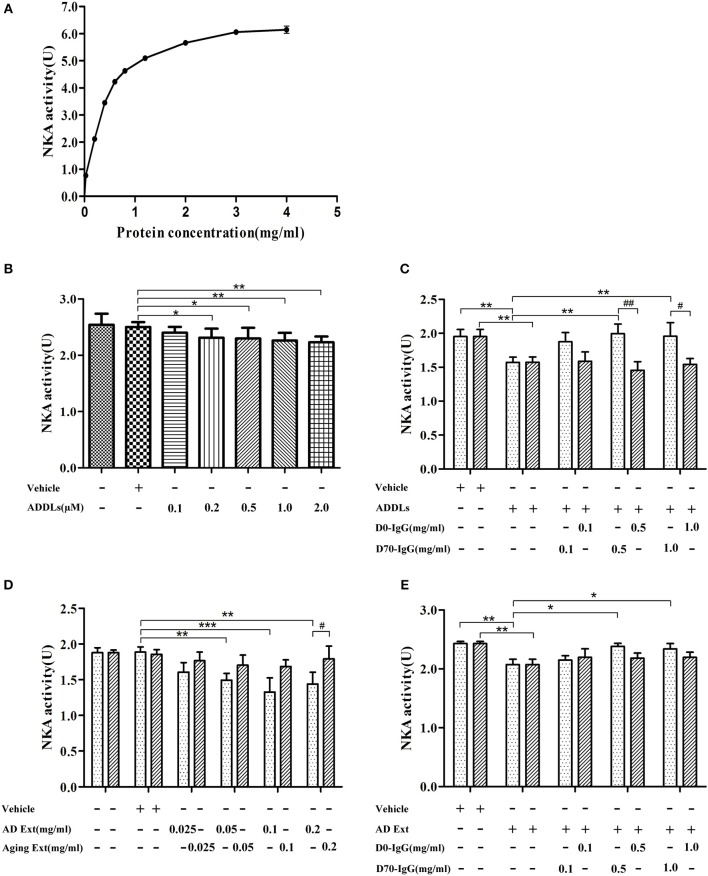
Antibody D70 to AβOs prevented AβOs inhibiting NKA activity of synaptosome *in vitro*. **(A)** Determination of NKA activity curve of synaptosome of mice by NKA enzyme Kit. The maximum activity of NKA was 6U, and the protein concentration corresponding to the 1/2Umax was 0.3 mg/ml (*n* = 3). **(B)** Artificial AβOs (ADDLs) inhibited mouse synaptosomal NKA activity (0.3 mg synaptosomal protein/ml) (*n* = 9). **(C)** AβO antibody (D70) prevented artificial AβOs (1 μM) from inhibiting synaptosomal NKA activity (*n* = 3). **(D)** Soluble Aβ extracts of human AD cerebral cortex inhibited NKA activities in mouse synaptosomes (*n* = 3). **(E)** AβO antibody (D70) prevented soluble Aβ extracts of human AD cerebral cortex inhibiting synaptosomal NKA activity (*n* = 3). Each value is expressed as mean ± SEM. *p < 0.05, **p < 0.01, ***p < 0.001, ^#^*p* < 0.05, ^*##*^*p* < 0.01.

Ligand blot demonstrated that soluble Aβ binds to α3-Na/K-ATPase protein, which is lost in PSD of human AD cortical tissues. However, the level of α3-Na/K-ATPase did not change in PSD of cortical tissues of the APP/PS1 mouse sampled at 6 months (data not shown), although it is reported that Na/K-ATPase activity is decreased in APP/PS1 mice (60). Here, we examined whether soluble oligomers of Aβ, which are formed in the early stages of AD, could inhibit Na/K-ATPase *in vitro*, and whether this inhibition could be prohibited by a polyclonal anti-Aβ antibody (4, 61, 62). Because we were uncertain which species could inhibit Na/K-ATPase, the polyclonal anti-AβOs antibody was tested. First, we tested the combined α1, 2-Na/K-ATPase activity in heart cell membrane and the combined α1, 2, 3-Na/K-ATPase activity in cortical synaptosomes. The protein amount at one-half the maximum activity of Na/K-ATPase was selected to demonstrate the inhibitory effect of AβOs on Na/K-ATPase activity (Figure 8). When the concentration of AβOs reached 100 nmol, AβOs significantly inhibited Na/K-ATPase activity in cortical synaptosomes (Figure 8). Interestingly, although AβOs did not show strong binding to α1, 2-Na/K-ATPase in ligand blot and co-PI, when concentration of AβOs was increased to 1 μM, AβOs could also significantly inhibit the activity of both (Supplementary Figure 1). In kidney cell membrane which mostly contained α1-Na/K-ATPase, the Na/K-ATPase also was inhibited by AβOs, which didn't show dose response (Supplementary Figure 2). These results indicated AβOs could interact with these Na/K-ATPase isoforms and inhibit their activities at nature condition, but that affinity to α1, α2-Na/K-ATPase could be not strong. Intriguingly, anti-soluble β-amyloid antibody can block AβOs inhibiting the activity of Na/K-ATPase (Figure 8, Supplementary Figures 1, 2).

## Discussion

### AβOs Bind to Na/K-ATPase, synGap, and Shank

Substantial evidence points to brain accumulation of soluble oligomers of amyloid-β peptide (AβOs) as a key early event in the pathogenesis of AD. We sought here to identify specific AβOs targeted proteins mediating synaptic dysfunction, which could potentially provide novel therapeutic targets for AD treatment.

**Figure 9:**
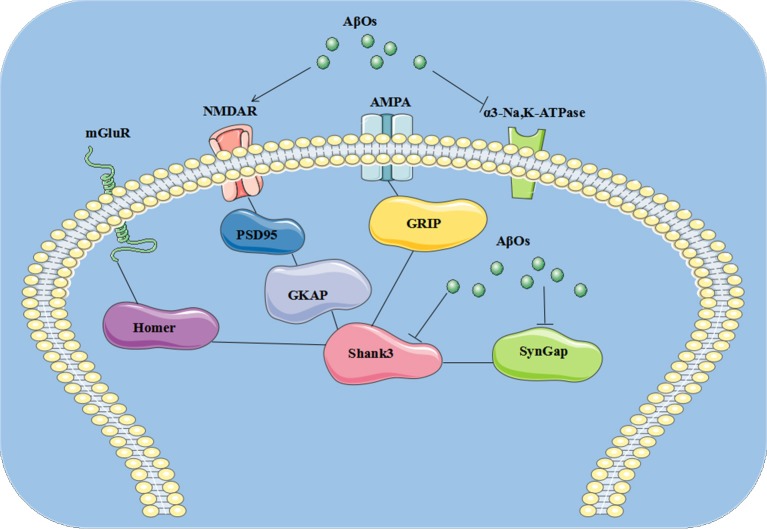
The pathological changes at synapse induced by AβOs. AβOs binding Na/K-ATPases could develop in early stages of AD brain, leading to synaptic dysfunction, while later stages could hypothetically result from disrupted synapse structure involving the PSD proteins synGap and Shank3.

In the present study, we identified three AβO binding proteins p100, p140, and p260, which we observed by ligand overlays in a previous paper (4), as α3-Na/K-ATPase, synGap, and Shank3. These three proteins are key components within the synapse needed to maintain synaptic structure and function (Table 3). α3-Na/K-ATPase on synaptic membranes regulate the neuron membrane potential for LTP formation; synGap and Shank in PSD organize and integrate and maintain the normal function of glutamate receptor signal network which underlying the memory formation (58). These results suggest oligomers of Aβ could target multiple synaptic proteins outside and inside the synapse, contributing to synaptic dysfunction in early stages of AD (Figure 9).

**Table 3:** Function of synaptic protein targets of amyloid beta oligomers.

**Protein name**	**Classification**	**Function**	**References**
α3-Na/K-ATPase	Neuronal receptor, transport proteins on cell membranes	A death target of Alzheimer patient amyloid-beta assembly, regulating neuron membrane potential	(49, 56, 63)
synGap	Synaptic GTPase activating protein	Maintaining signal network of glutamate receptor	(60, 64)
Shank3	Scaffold protein of PSD	Promoting the formation of excitatory synapses and the development of dendritic spines, maintain the function of glutamate receptors	(65, 66)

### Na/K-ATPase, synGap, and Shank3 Proteins Are Altered in PSD of Human AD Brain

Synaptic loss is the strongest anatomical correlate to the degree of clinical impairment, although the molecular pathological mechanism of synaptic loss remains elusive. In human AD postmodern tissues, Shank3, NMDA receptor, and AMPA receptor are lost in the PSD fraction. From proteomics quantitative data obtained here, synGap and α3-Na/K-ATPase proteins are also seen to be lost in the PSD fraction of AD cortical tissues (Table 2, Figure 5) and (52).

SynGap at the excitatory synapse is integrated to PSD95, Shank, and Ca2^+^/CaM Kinase II, a core complex of signaling proteins associated with the NMDA receptor in the PSD which influence several neuronal processes (67). In this core, synGap (p140) and Shank (p260) were clearly decreased (4), along with PSD95, in human AD brain (68). The increased AβOs in AD brain (4) may likely be involved in inducing loss of these proteins, as in neuronal cultures, oligomers of β-amyloid can activate NMDA receptor leading to PSD95 degradation (64). Binding of AβOs to synGap (p140) could contribute to this degradation, although more experiments are needed to investigate this speculation. Exogenous oligomers of β-amyloid are typically bound to the surfaces of neurons, close to NMDA receptors (7, 23), and NMDA receptors appear to play an indirect role in AβO binding. NMDA receptor and synGap are associated and closed, AβO puncta were found to co-localize with synGap, AβOs could be internalized for a long term incubation and bind to synGap and Shank3 (69, 70). However, these AβOs puncta were also shown at synapse of neuron with synGap (–/–) (data not shown), which suggests AβOs could have multiple targets, the precise pathogenic mechanism of AβO at puncta need to be further established.

### AβOs Attach to Na/K-ATPases at the Synapse

Multiple lines of evidence implicate soluble oligomeric assemblies of Aβ peptides in the pathogenesis of Alzheimer's disease. Here, we used human AD frontal cortical tissues to isolate synaptosome fractions; these synaptosomes showed high abundance of oligomers of Aβ, which can be identified by antibody A11 (Figure 6), and α3-Na/K-ATPase (Figure 6). Furthermore, synaptic oligomers of Aβ and Na/K-ATPases are pulled down together, which suggests that synaptic oligomers of β peptide may directly attack α3-Na/K-ATPase (Figure 6).

α3-Na/K-ATPase has been reported as neuronal receptor (56). ASPD and artificial AβOs can bind to α3-Na/K-ATPase, which suggest various species of Aβ may directly and commonly attack α3-Na/K-ATPase in AD brains.

In addition to the above analysis, various tissues of mice were used to confirm oligomers of Aβ binding to Na/K-ATPase. When mice heart and kidney cell membranes, and cortical synaptosomes were analyzed by antibody to α1-, α2-, α3-Na/K-ATPase isoforms, cortical synaptosomes contained these three isoforms, heart membrane contained α1-, α2- isoforms, kidney only contained α1-isoform. Results were confirmed by α3-Na/K-ATPase protein in different distributions in various tissues; α1-Na/K-ATPase was expressed in the heart of guinea pig, dog, sheep, α3 only in dog ventricle (54). In oligomers of Aβ ligand blot experiment, oligomers of Aβ only bound to p100 in cortical synaptosome, not to p100 protein such as α1- and α2-Na/K-ATPase in heart (Figure 7) which confirm oligomers of Aβ binding to p100 which is α3-Na/K-ATPase in our previous publication (4), these binding was identical to that ASPD and AβOs binding to α3-Na, K-ATPase (38, 46).

Meanwhile, we found α3-Na/K-ATPase protein was lost at the PSD of human AD frontal cortex, which are late stage of AD (Figure 5), and this protein loss did not exist in PSD of APP/PS1 mice brains (data not shown). However, the soluble AβOs, extracted from 3 × Tg mice from as early as 4 months old, could bind to p100 (α3-Na/K-ATPase) in AβO ligand blot (Figure 7D), and the Na/K-ATPase activity loss was demonstrated in APP/PS1 mice brains, which suggests that oligomers of Aβ may inhibit activity of Na/K-ATPase in early stages of AD. We used oligomers of Aβ, as early pathological species of Aβ, to test whether they can selectively inhibit α3-Na/K-ATPase activity. Mice cortical synaptosome, heart cell membrane and kidney cell membrane were used to test bioactivity of oligomers of Aβ. Results showed that oligomers of Aβ can inhibit all these kinds of Na/K-ATPase activity, and did not show selectively inhibit α3-Na/K-ATPase activity, which suggests that oligomers of Aβ may inhibit all α1,2,3-Na/K-ATPase activity. However, we did not find soluble Aβ directly binding to α1,2-Na/K-ATPase in co-IP and ligand blot.

Since Na/K-ATPase activity is lost during the development of AD, restoration of Na/K-ATPase activity may protect synaptic function. Until now, the specific toxic species of Aβ responsible for this damage remains elusive. The polyclonal antibody to soluble Aβ was used to block AβOs toxicity to Na/K-ATPase; we found this antibody to restore Na/K-ATPase activity which was inhibited by oligomers of Aβ, suggesting that antibodies to soluble Aβ may protect Na/K-ATPase activity in AD brain, and even in AD peripheral tissues.

### Possible Effects of AβOs on Na/K-ATPase and Synaptic Function

Na/K-ATPase is critical in the maintenance of resting membrane potential, restoration of membrane potential following neuronal depolarization, as well as maintenance of osmotic balance and cell volume (71). The oligomers of Aβ are formed earlier than plaques of Aβ (6). Synaptic proteins including α3-Na/K-ATPase, synGap, and Shank3 did not show changes at early stages of AD in mice brains (data not shown), however oligomers of Aβ can significantly inhibit Na/K-ATPase activity *in vitro* (Figure 8) (60) and *in vivo* (65), which suggests that oligomers of Aβ may regulate Na/K-ATPase activity in AD brain. Inhibition of the Na/K-ATPase not only decreases intracellular K^+^ but also increases intracellular Ca2^+^; the latter of which may trigger neuronal toxicity (72).

Na/K-ATPase plays a critical role in maintaining normal neuronal functions, in which Na/K-ATPase activity is regulated by specific Lyn tyrosine kinases via a protein-protein mechanism that may play a role in apoptosis (73). Lyn also regulates the AMPA receptor pathway for generating intracellular signals from the cell surface to the nucleus through the Lyn-MAPK pathway, which may contribute to synaptic plasticity and transforming short-term plasticity to long-term plasticity (74). Na/K-ATPase and NMDA play important roles in the formation of memory in the hippocampus, suggesting potential cross-talk between Na/K-ATPase and the NMDA receptor (75).

### Na/K-ATPase and Alzheimer's Disease

Na/K-ATPase may play an important role especially in early stages of AD development. While synaptic protein levels remain within normal ranges, the activity of Na/K-ATPase is inhibited by soluble aggregates of Aβ, which may lead to synaptic dysfunction (38, 46, 60).

In early stages of AD, the slight impairment of Na/K-ATPase activity, inhibited by soluble aggregates of Aβ (Figure 8), may amplify the disruption of K^+^ homeostasis, thus activating the apoptotic cascade and causing substantial neuronal injury (76). Lately, the protein abundance of Na/K-ATPase and neuron specific Na/K-ATPase α3 isoform was significantly reduced in the PSD fraction of AD brains (Figure 5). Na/K-ATPases are selectively impaired in AD brains, which may cause an excitotoxic cellular response and result in neuronal death (77).

Overall Na/K-ATPase activity was decreased in the amyloid-containing hippocampi of the APP+PS1 mice, but not in the amyloid-free cerebellum, which correlates with endogenous AβOs in human frontal cortex, but not in cerebellum. In addition, cerebral Na/K-ATPase activity can be directly inhibited by high concentrations of soluble Aβ (60).

Excitotoxicity exists in the AD brain. Ouabain, a selective inhibitor of Na/K-ATPase, elicits greater than appropriate glutamate-receptor overstimulation resulting in excitotoxicity. Na pump isoforms in neurons differ in their physiological significance, and the brain type isoform plays an important role in restoring concentration gradients of Na^+^ and K^+^ after neuronal excitation (63, 78). The mutation of Na/K-ATPase leads to reduction of this enzyme activity and could cause epilepsy (79). Interestingly, patients with late-onset epilepsy of unknown origin have a high prevalence of abnormal Aβ1-42 and progression to AD dementia (80).

### AβOs Attach to Intracellular and Extracellular Components of Synapses

The toxicity of the protein aggregates will engage in a multitude of aberrant interactions with various cellular components, such as protein receptors, soluble proteins, and other targets, which have the significant potential to cause cellular dysfunction (81).

Multiple lines of evidence implicate soluble oligomers of Aβ in the pathogenesis of AD, which have been reported to interact with the phospholipid bilayers of the cell membrane, NMDA, and AMPA receptors, the metabotropic glutamate receptor 5, the insulin receptor, the nicotinic acetylcholine receptor α7-nAcChR, PrP, and other cellular components and membrane receptors (82). We found oligomers of Aβ could target Na/K-ATPase, synGap, and shank3 (4, 38, 46) to modify NMDA receptor signaling pathways, thus contributing to synaptic dysfunction in AD. Perhaps this helps to explain why memantine, an NMDA receptor antagonist, serves as symptomatic therapy for AD. AβOs could induce the pathological changes at synapse, which is summarized in cartoon (Figure 9).

## Conclusion

In the present study, we identified three AβO binding proteins as α3-Na/K-ATPase, synGap, and Shank3. These proteins are key components within the synapse needed to maintain synaptic structure and function. Soluble assemblies of Aβ peptide binding Na/K-ATPases could develop in early stages of AD brain, leading to synaptic dysfunction, while later stages could hypothetically result from disrupted synapse structure involving the PSD proteins synGap and Shank3. This discovery provides new possible therapeutic targets for AD treatment at early stages by antibodies or other agents (Figure 9).

## Data Availability Statement

The datasets used and/or analyzed during the current study are available from the corresponding author on reasonable.

## Ethics Statement

The study was approved by the Institutional Animal Care and Use Committee of Nanjing University of Chinese Medicine, Northwestern University, and Drexel University College of Medicine.

## Author Contributions

WK and YG contributed study concept and design. YD also contributed study design. YD, JZ, XZ, SW, KV, FC, and YG acquired, analyzed, or interpreted data. YZ participated in membrane preparation and AβOs preparation. CL collected human brain tissues and participated in the data analysis. WK, YG, and KV drafted and revised the manuscript. All authors read and approved the final manuscript.

### Conflict of Interest

The authors declare that the research was conducted in the absence of any commercial or financial relationships that could be construed as a potential conflict of interest.

## Supplementary Material

Supplementary Figure 1 Antibody D70 to AβOs prevented AβOs inhibiting NKA activity of heart cell membrane *in vitro*. **(A)** Determination of NKA activity curve of heart cell membrane of mice by NKA enzyme Kit. The maximum enzyme activity was 4.0 U, and the protein concentration corresponding to the 1/2 U max was 0.25 mg/ml (*n* = 3). **(B)** Artificial ADDLs inhibited NKA activities in heart cell membrane of mice at 0.25 mg/ml in reaction system (*n* = 9). **(C)** Antibody D70 to oligomers of Aβ prevent artificial oligomers of Aβ at 1 μM inhibiting activities of NKA of heart cell membrane (*n* = 3). **(D)** Soluble Aβ extracted from the cerebral cortex of human AD inhibited activities of NKA in heart membrane of mice (*n* = 3). **(E)** Antibody D70 to oligomers of Aβ prevent soluble Aβ extracted from the cerebral cortex of human AD inhibiting activities of NKA in heart cell membrane of mice (*n* = 3). Each value is expressed as mean ± SEM. ^*^*p* < 0.05, ^**^*p* < 0.01, ^***^*p* < 0.001, ^#^*p* < 0.05, ^*##*^*p* < 0.01.

Supplementary Figure 2 Antibody D70 to AβOs prevented AβOs inhibiting NKA activity of kidney cell membrane *in vitro*. **(A)** Determination of NKA activity curve of kidney cell membrane of mice by NKA enzyme Kit. The maximum enzyme activity was 7.3 U, and the protein concentration corresponding to the 1/2 U max was 0.25 mg/ml (*n* = 3). **(B)** Artificial ADDLs inhibited NKA activities in kidney cell membrane of mice at 0.25 mg/ml in reaction system (*n* = 9). **(C)** Antibody D70 to oligomers of Aβ prevent artificial oligomers of Aβ at 1 μM inhibiting activities of NKA of kidney cell membrane (*n* = 3). **(D)** Soluble Aβ extracted from the cerebral cortex of human AD inhibited activities of NKA in kidney membrane of mice (*n* = 3). **(E)** Antibody D70 to oligomers of Aβ prevent soluble Aβ extracted from the cerebral cortex of human AD inhibiting activities of NKA in kidney cell membrane of mice (*n* = 3). Each value is expressed as mean ± SEM. ^*^*p* < 0.05, ^**^*p* < 0.01, ^***^*p* < 0.001, ^#^*p* < 0.05.
