# Supplementary figures and images for: Amyloid Beta Oligomers Target to Extracellular and Intracellular Neuronal Synaptic Proteins in Alzheimer's Disease
Source: Front Neurol. 2019 Nov 1;10:1140. doi: 10.3389/fneur.2019.01140 (PMC6838211; doi:10.3389/fneur.2019.01140)

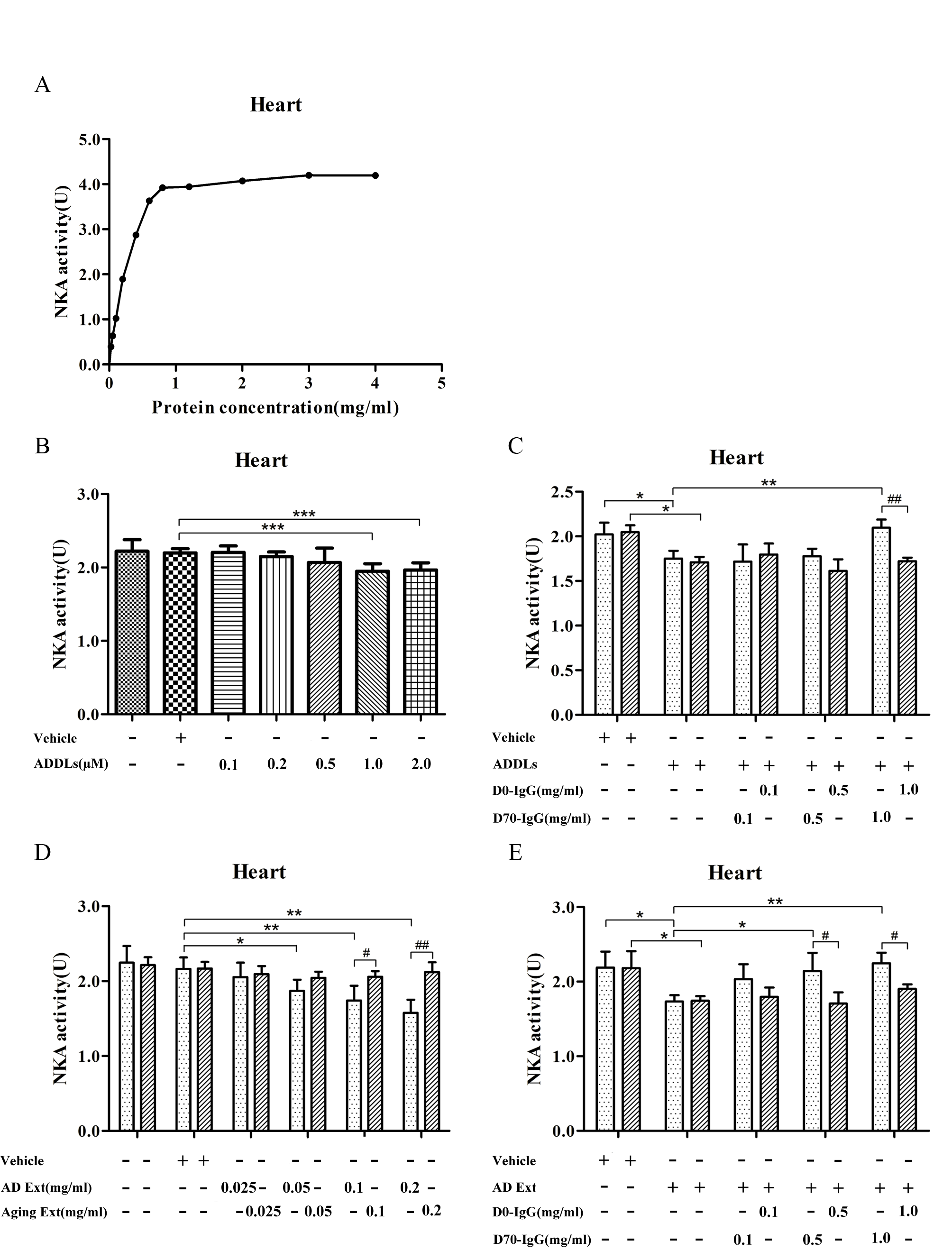

Supplement: Supplementary Figure 1 — Antibody D70 to AβOs prevented AβOs inhibiting NKA activity of heart cell membrane in vitro. (A) Determination of NKA activity curve of heart cell membrane of mice by NKA enzyme Kit. The maximum enzyme activity was 4.0 U, and the protein concentration corresponding to the 1/2 U max was 0.25 mg/ml (n = 3). (B) Artificial ADDLs inhibited NKA activities in heart cell membrane of mice at 0.25 mg/ml in reaction system (n = 9). (C) Antibody D70 to oligomers of Aβ prevent artificial oligomers of Aβ at 1 μM inhibiting activities of NKA of heart cell membrane (n = 3). (D) Soluble Aβ extracted from the cerebral cortex of human AD inhibited activities of NKA in heart membrane of mice (n = 3). (E) Antibody D70 to oligomers of Aβ prevent soluble Aβ extracted from the cerebral cortex of human AD inhibiting activities of NKA in heart cell membrane of mice (n = 3). Each value is expressed as mean ± SEM. *p < 0.05, **p < 0.01, ***p < 0.001, #p < 0.05, ##p < 0.01. [file Image_1.TIF]

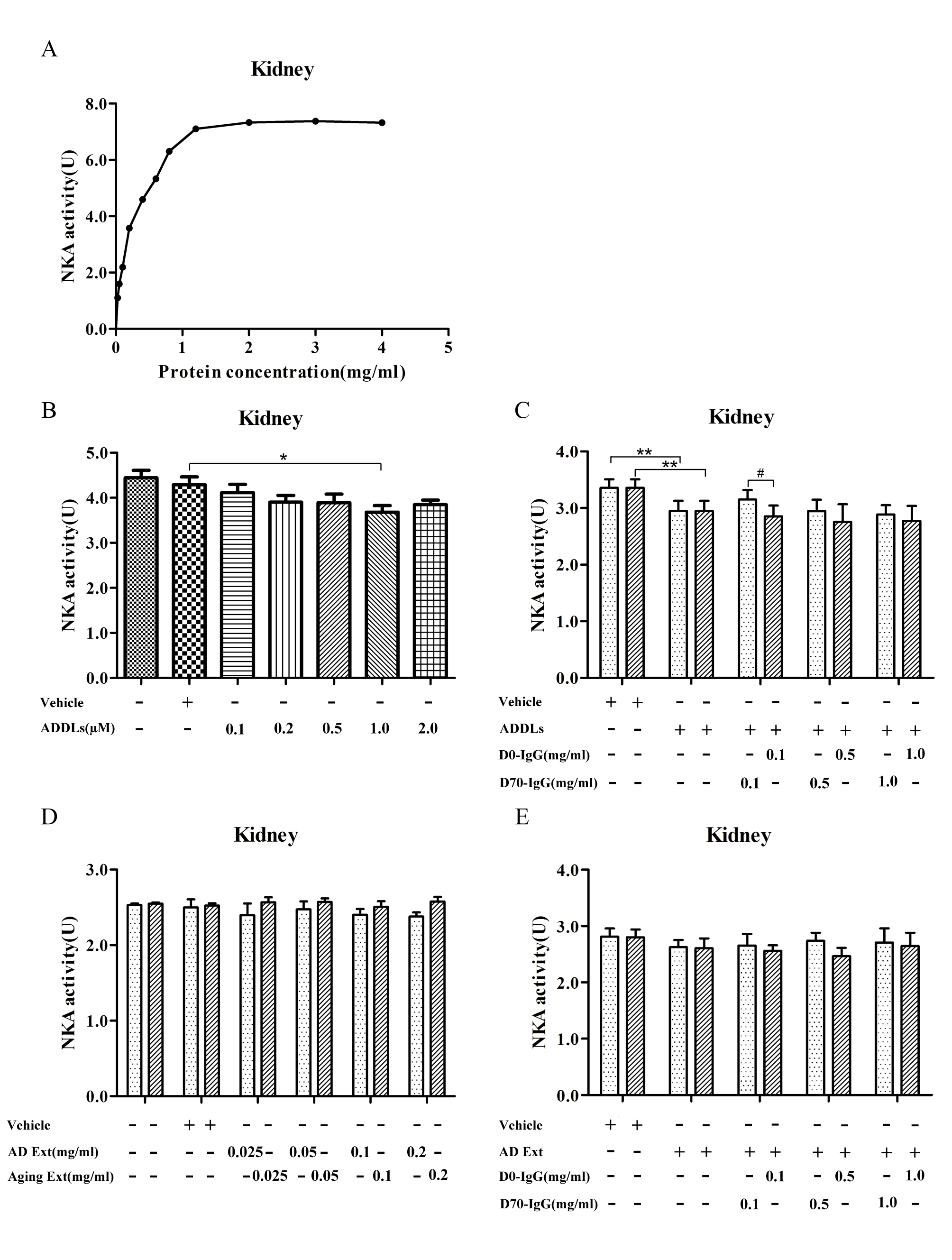

Supplement: Supplementary Figure 2 — Antibody D70 to AβOs prevented AβOs inhibiting NKA activity of kidney cell membrane in vitro. (A) Determination of NKA activity curve of kidney cell membrane of mice by NKA enzyme Kit. The maximum enzyme activity was 7.3 U, and the protein concentration corresponding to the 1/2 U max was 0.25 mg/ml (n = 3). (B) Artificial ADDLs inhibited NKA activities in kidney cell membrane of mice at 0.25 mg/ml in reaction system (n = 9). (C) Antibody D70 to oligomers of Aβ prevent artificial oligomers of Aβ at 1 μM inhibiting activities of NKA of kidney cell membrane (n = 3). (D) Soluble Aβ extracted from the cerebral cortex of human AD inhibited activities of NKA in kidney membrane of mice (n = 3). (E) Antibody D70 to oligomers of Aβ prevent soluble Aβ extracted from the cerebral cortex of human AD inhibiting activities of NKA in kidney cell membrane of mice (n = 3). Each value is expressed as mean ± SEM. *p < 0.05, **p < 0.01, ***p < 0.001, #p < 0.05. [file Image_2.TIF]
